# PubMed related articles: a probabilistic topic-based model for content similarity

**DOI:** 10.1186/1471-2105-8-423

**Published:** 2007-10-30

**Authors:** Jimmy Lin, W John Wilbur

**Affiliations:** 1 College of Information Studies, University of Maryland, College Park, Maryland, USA; 2 National Center for Biotechnology Information, National Library of Medicine, Bethesda, Maryland, USA

## Abstract

**Background:**

We present a probabilistic topic-based model for content similarity called *pmra *that underlies the related article search feature in PubMed. Whether or not a document is about a particular topic is computed from term frequencies, modeled as Poisson distributions. Unlike previous probabilistic retrieval models, we do not attempt to estimate relevance–but rather our focus is "relatedness", the probability that a user would want to examine a particular document given known interest in another. We also describe a novel technique for estimating parameters that does not require human relevance judgments; instead, the process is based on the existence of MeSH ^® ^in MEDLINE ^®^.

**Results:**

The *pmra *retrieval model was compared against *bm25*, a competitive probabilistic model that shares theoretical similarities. Experiments using the test collection from the TREC 2005 genomics track shows a small but statistically significant improvement of *pmra *over *bm25 *in terms of precision.

**Conclusion:**

Our experiments suggest that the *pmra *model provides an effective ranking algorithm for related article search.

## Background

**Figure 1:**
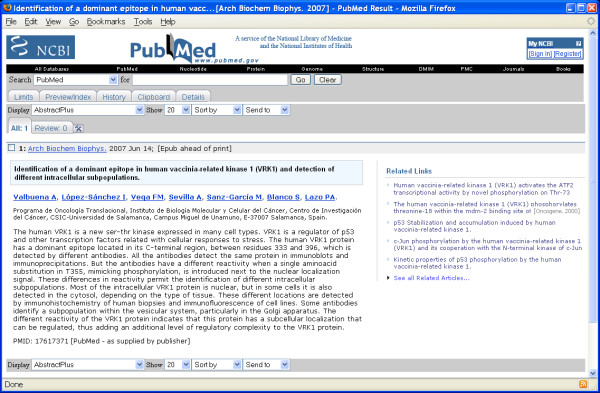
A typical view in the PubMed search interface showing an abstract in detail. The "Related Links" panel on the right is populated with titles of articles that may be of interest.

This article describes the retrieval model behind the related article search functionality in PubMed [1]. Whenever the user examines a MEDLINE citation in detail, a panel to the right of the abstract text is automatically populated with titles of articles that may also be of interest (see Figure 1). We describe *pmra*, the topic-based content similarity model that underlies this feature.

There is evidence to suggest that related article search is a useful feature. Based on PubMed query logs gathered during a one-week period in June 2007, we observed approximately 35 million page views across 8 million browser sessions. Of those sessions, 63% consisted of a single page view–representing bots and direct access into MEDLINE (e.g., from an embedded link or another search engine). Of all sessions in our data set, approximately 2 million include at least one PubMed search query and at least one view of an abstract–this figure roughly quantifies actual searches. About 19% of these involve at least one click on a related article. In other words, roughly a fifth of all non-trivial user sessions contain at least one invocation of related article search. In terms of overall frequency, approximately five percent of all page views in these non-trivial sessions were generated from clicks on related article links. More details can be found in [2].

We evaluate the *pmra *retrieval model with the test collection from the TREC 2005 genomics track. A test collection is a standard laboratory tool for evaluating retrieval systems, and it consists of three major components:

• a corpus–a collection of documents on which retrieval is performed,

• a set of information needs–written statements describing the desired information, which translate into queries to the system, and

• relevance judgments–records specifying the documents that should be retrieved in response to each information need (typically, these are gathered from human assessors in large-scale evaluations [3]).

The use of test collections to assess the performance of retrieval algorithms is a well-established methodology in the information retrieval (IR) literature, dating back to the Cranfield experiments in the 60's [4]. These tools enable rapid, reproducible experiments in a controlled setting without requiring users.

The *pmra *model is compared against *bm25 *[5,6], a competitive probabilistic model that shares theoretical similarities with *pmra*. On test data from the TREC 2005 genomics track, we observe a small but statistically significant improvement in terms of precision.

Before proceeding, a clarification on terminology: although MEDLINE records contain only abstract text and associated bibliographic information, PubMed provides access to the full text articles (if available). Thus, it is not inaccurate to speak of searching for articles, even though the search itself is only performed on information in MEDLINE. Throughout this work, we use "document" and "article" interchangeably.

### 1.1 Formal Model

We formalize the related document search problem as follows: given a document that the user has indicated interest in, the system task is to retrieve other documents that the user may also want to examine. Since this activity generally occurs in the context of broader information-seeking behaviors, relevance can serve as one indicator of interest, i.e., retrieve other relevant documents. However, we think of the problem in broader terms: other documents may be interesting because they discuss similar topics, share the same citations, provide general background, lead to interesting hypotheses, etc.

To constrain this problem, we assume in our theoretical model that documents of interest are similar in terms of the topics or concepts that they are *about*; in the case of MEDLINE citations, we limit ourselves to the article title and abstract (the deployed algorithm in PubMed also takes advantage of MeSH terms, which we do not discuss here). Following typical assumptions in information retrieval [7], we wish to rank documents (MEDLINE citations, in our case) based on the probability that the user will want to see them. Thus, our *pmra *retrieval model focuses on estimating *P*(*c*|*d*), the probability that the user will find document *c *interesting given expressed interest in document *d*.

Let us begin by decomposing documents into mutually-exclusive and exhaustive "topics" (denoted by the set {*s*_1_...*s*_*N*_}). Assuming that the relatedness of documents is mediated through topics, we get the following:

P(c|d)=∑j=1NP(c|sj)P(sj|d)
 MathType@MTEF@5@5@+=feaafiart1ev1aaatCvAUfKttLearuWrP9MDH5MBPbIqV92AaeXatLxBI9gBaebbnrfifHhDYfgasaacH8akY=wiFfYdH8Gipec8Eeeu0xXdbba9frFj0=OqFfea0dXdd9vqai=hGuQ8kuc9pgc9s8qqaq=dirpe0xb9q8qiLsFr0=vr0=vr0dc8meaabaqaciaacaGaaeqabaqabeGadaaakeaacqWGqbaucqGGOaakcqWGJbWycqGG8baFcqWGKbazcqGGPaqkcqGH9aqpdaaeWbqaaiabdcfaqjabcIcaOiabdogaJjabcYha8jabdohaZnaaBaaaleaacqWGQbGAaeqaaOGaeiykaKIaemiuaaLaeiikaGIaem4Cam3aaSbaaSqaaiabdQgaQbqabaGccqGG8baFcqWGKbazcqGGPaqkaSqaaiabdQgaQjabg2da9iabigdaXaqaaiabd6eaobqdcqGHris5aaaa@4CC1@

Expanding *P*(*s*_*j*_|*d*) by Bayes' Theorem, we get:

P(c|d)=∑j=1NP(c|sj)P(d|sj)P(sj)∑j=1NP(d|sj)P(sj)
 MathType@MTEF@5@5@+=feaafiart1ev1aaatCvAUfKttLearuWrP9MDH5MBPbIqV92AaeXatLxBI9gBaebbnrfifHhDYfgasaacH8akY=wiFfYdH8Gipec8Eeeu0xXdbba9frFj0=OqFfea0dXdd9vqai=hGuQ8kuc9pgc9s8qqaq=dirpe0xb9q8qiLsFr0=vr0=vr0dc8meaabaqaciaacaGaaeqabaqabeGadaaakeaacqWGqbaucqGGOaakcqWGJbWycqGG8baFcqWGKbazcqGGPaqkcqGH9aqpdaWcaaqaamaaqadabaGaemiuaaLaeiikaGIaem4yamMaeiiFaWNaem4Cam3aaSbaaSqaaiabdQgaQbqabaGccqGGPaqkcqWGqbaucqGGOaakcqWGKbazcqGG8baFcqWGZbWCdaWgaaWcbaGaemOAaOgabeaakiabcMcaPiabdcfaqjabcIcaOiabdohaZnaaBaaaleaacqWGQbGAaeqaaOGaeiykaKcaleaacqWGQbGAcqGH9aqpcqaIXaqmaeaacqWGobGta0GaeyyeIuoaaOqaamaaqadabaGaemiuaaLaeiikaGIaemizaqMaeiiFaWNaem4Cam3aaSbaaSqaaiabdQgaQbqabaGccqGGPaqkcqWGqbaucqGGOaakcqWGZbWCdaWgaaWcbaGaemOAaOgabeaakiabcMcaPaWcbaGaemOAaOMaeyypa0JaeGymaedabaGaemOta4eaniabggHiLdaaaaaa@677D@

Since we are only concerned about the ranking of documents, the denominator can be safely ignored since it is independent of *c*. Thus, we arrive at the following criteria for ranking documents:

P(c|d)∝∑j=1NP(c|sj)P(d|sj)P(sj)
 MathType@MTEF@5@5@+=feaafiart1ev1aaatCvAUfKttLearuWrP9MDH5MBPbIqV92AaeXatLxBI9gBaebbnrfifHhDYfgasaacH8akY=wiFfYdH8Gipec8Eeeu0xXdbba9frFj0=OqFfea0dXdd9vqai=hGuQ8kuc9pgc9s8qqaq=dirpe0xb9q8qiLsFr0=vr0=vr0dc8meaabaqaciaacaGaaeqabaqabeGadaaakeaacqWGqbaucqGGOaakcqWGJbWycqGG8baFcqWGKbazcqGGPaqkcqGHDisTdaaeWbqaaiabdcfaqjabcIcaOiabdogaJjabcYha8jabdohaZnaaBaaaleaacqWGQbGAaeqaaOGaeiykaKIaemiuaaLaeiikaGIaemizaqMaeiiFaWNaem4Cam3aaSbaaSqaaiabdQgaQbqabaGccqGGPaqkcqWGqbaucqGGOaakcqWGZbWCdaWgaaWcbaGaemOAaOgabeaakiabcMcaPaWcbaGaemOAaOMaeyypa0JaeGymaedabaGaemOta4eaniabggHiLdaaaa@5318@

Rephrased in prose, *P*(*c*|*s*_*j*_) is the probability that a user would want to see *c *given an interest in topic *s*_*j*_, and similarly for *P*(*d*|*s*_*j*_). Thus, the degree to which two documents are related can be computed by the product of these two probabilities and the prior probability on the topic *P*(*s*_*j*_), summed across all topics.

Thus far, we have not addressed the important question of what a topic actually is. For computational tractability, we make the simplifying assumption that each term in a document represents a topic (that is, each term conveys an idea or concept). Thus, the "aboutness" of a document (i.e., what topics the document discusses) is conveyed through the terms in the document. As with most retrieval models, we assume single-word terms, as opposed to potentially complex multi-word concepts. This satisfies our requirement that the set of topics be exhaustive and mutually-exclusive.

From this starting point, we leverage previous work in probabilistic retrieval models based on Poisson distributions (e.g., [6,8,9]). A Poisson distribution characterizes the probability of a specific number of events occurring in a fixed period of time if these events occur with a known average rate. The underlying assumption is a generative model of document content: let us suppose that an author uses a particular term with constant probability, and that documents are generated as a sequence of terms. A Poisson distribution specifies the probability that we would observe the term *n *times in a document. Obviously, this does not accurately reflect how content is actually produced–nevertheless, this simple model has served as the starting point for many effective retrieval algorithms.

This content model also assumes that each term occurrence is independent. Although in reality term occurrences are *not *independent–for example, observing the term "breast" in a document makes the term "cancer" more likely to also be observed–such a simplification makes the problem computationally tractable. This is commonly known as the term-independence assumption and dates back to the earliest days of information retrieval research [10]. See [11] for recent work that attempts to introduce term dependencies into retrieval algorithms.

Building on this, we invoke the concept of *eliteness*, which is closely associated with probabilistic IR models [8]. A given document *d *can be *about *a particular topic *s*_*i *_or not. Following standard definitions, in the first case we say that the term *t*_*i *_(representing the topic *s*_*i*_) is *elite *for document *d *(and not elite in the second case).

Let us further assume, as others have before, that elite terms and non-elite terms are used with different frequencies. That is, if the author intends to convey topic *s*_*i *_in a document, the author will use term *t*_*i *_with a certain probability (elite case); if the document is not about *s*_*i*_, the author will use term *t*_*i *_with a different (presumably smaller) probability. We can characterize the observed frequency of a term by a Poisson distribution, defined by a single parameter (the mean), which in our model is different for the elite and non-elite cases.

Thus, we wish to compute *P*(*E*|*k*)–the probability that a document is *about *a topic, given that we observed its corresponding term *k *times in the document. By Bayes' rule:

P(E|k)=P(k|E)P(E)P(k|E)P(E)+P(k|E¯)P(E¯)
 MathType@MTEF@5@5@+=feaafiart1ev1aaatCvAUfKttLearuWrP9MDH5MBPbIqV92AaeXatLxBI9gBaebbnrfifHhDYfgasaacH8akY=wiFfYdH8Gipec8Eeeu0xXdbba9frFj0=OqFfea0dXdd9vqai=hGuQ8kuc9pgc9s8qqaq=dirpe0xb9q8qiLsFr0=vr0=vr0dc8meaabaqaciaacaGaaeqabaqabeGadaaakeaacqWGqbaucqGGOaakcqWGfbqrcqGG8baFcqWGRbWAcqGGPaqkcqGH9aqpdaWcaaqaaiabdcfaqjabcIcaOiabdUgaRjabcYha8jabdweafjabcMcaPiabdcfaqjabcIcaOiabdweafjabcMcaPaqaaiabdcfaqjabcIcaOiabdUgaRjabcYha8jabdweafjabcMcaPiabdcfaqjabcIcaOiabdweafjabcMcaPiabgUcaRiabdcfaqjabcIcaOiabdUgaRjabcYha8jqbdweafzaaraGaeiykaKIaemiuaaLaeiikaGIafmyrauKbaebacqGGPaqkaaaaaa@55D2@

=(1+P(k|E¯)P(E¯)P(k|R)P(E))−1
 MathType@MTEF@5@5@+=feaafiart1ev1aaatCvAUfKttLearuWrP9MDH5MBPbIqV92AaeXatLxBI9gBaebbnrfifHhDYfgasaacH8akY=wiFfYdH8Gipec8Eeeu0xXdbba9frFj0=OqFfea0dXdd9vqai=hGuQ8kuc9pgc9s8qqaq=dirpe0xb9q8qiLsFr0=vr0=vr0dc8meaabaqaciaacaGaaeqabaqabeGadaaakeaacqGH9aqpdaqadaqaaiabigdaXiabgUcaRmaalaaabaGaemiuaaLaeiikaGIaem4AaSMaeiiFaWNafmyrauKbaebacqGGPaqkcqWGqbaucqGGOaakcuWGfbqrgaqeaiabcMcaPaqaaiabdcfaqjabcIcaOiabdUgaRjabcYha8jabdkfasjabcMcaPiabdcfaqjabcIcaOiabdweafjabcMcaPaaaaiaawIcacaGLPaaadaahaaWcbeqaaiabgkHiTiabigdaXaaaaaa@48E7@

Next, we must compute the two probabilities *P*(*k*|*E*) and *P*(*k*|E¯
 MathType@MTEF@5@5@+=feaafiart1ev1aaatCvAUfKttLearuWrP9MDH5MBPbIqV92AaeXatLxBI9gBaebbnrfifHhDYfgasaacH8akY=wiFfYdH8Gipec8Eeeu0xXdbba9frFj0=OqFfea0dXdd9vqai=hGuQ8kuc9pgc9s8qqaq=dirpe0xb9q8qiLsFr0=vr0=vr0dc8meaabaqaciaacaGaaeqabaqabeGadaaakeaacuWGfbqrgaqeaaaa@2DD7@). As discussed above, we model the two as Poisson distributions. For the elite case, the distribution is defined by the parameter *λ*, for the non-elite case, the parameter *μ*:

P(k|E)=λke−λk!
 MathType@MTEF@5@5@+=feaafiart1ev1aaatCvAUfKttLearuWrP9MDH5MBPbIqV92AaeXatLxBI9gBaebbnrfifHhDYfgasaacH8akY=wiFfYdH8Gipec8Eeeu0xXdbba9frFj0=OqFfea0dXdd9vqai=hGuQ8kuc9pgc9s8qqaq=dirpe0xb9q8qiLsFr0=vr0=vr0dc8meaabaqaciaacaGaaeqabaqabeGadaaakeaacqWGqbaucqGGOaakcqWGRbWAcqGG8baFcqWGfbqrcqGGPaqkcqGH9aqpdaWcaaqaaGGaciab=T7aSnaaCaaaleqabaGaem4AaSgaaOGaemyzau2aaWbaaSqabeaacqGHsislcqWF7oaBaaaakeaacqWGRbWAcqGGHaqiaaaaaa@3E2F@

P(k|E¯)=μke−μk!
 MathType@MTEF@5@5@+=feaafiart1ev1aaatCvAUfKttLearuWrP9MDH5MBPbIqV92AaeXatLxBI9gBaebbnrfifHhDYfgasaacH8akY=wiFfYdH8Gipec8Eeeu0xXdbba9frFj0=OqFfea0dXdd9vqai=hGuQ8kuc9pgc9s8qqaq=dirpe0xb9q8qiLsFr0=vr0=vr0dc8meaabaqaciaacaGaaeqabaqabeGadaaakeaacqWGqbaucqGGOaakcqWGRbWAcqGG8baFcuWGfbqrgaqeaiabcMcaPiabg2da9maalaaabaacciGae8hVd02aaWbaaSqabeaacqWGRbWAaaGccqWGLbqzdaahaaWcbeqaaiabgkHiTiab=X7aTbaaaOqaaiabdUgaRjabcgcaHaaaaaa@3E4B@

After further algebraic manipulation, we get the expression in Equation 8. Since there are differences in length between documents in the same collection, we account for this by introducing *l*, the length of the document in words. Previous research has shown that document length normalization plays an important role in retrieval performance (e.g., [12]), since longer documents are likely to have more query terms *a priori*. Finally, we define the parameter *η *= *P*(E¯
 MathType@MTEF@5@5@+=feaafiart1ev1aaatCvAUfKttLearuWrP9MDH5MBPbIqV92AaeXatLxBI9gBaebbnrfifHhDYfgasaacH8akY=wiFfYdH8Gipec8Eeeu0xXdbba9frFj0=OqFfea0dXdd9vqai=hGuQ8kuc9pgc9s8qqaq=dirpe0xb9q8qiLsFr0=vr0=vr0dc8meaabaqaciaacaGaaeqabaqabeGadaaakeaacuWGfbqrgaqeaaaa@2DD7@)/*P*(*E*).

P(E|k)=(1+η(μλ)ke−(μ−λ)l)−1
 MathType@MTEF@5@5@+=feaafiart1ev1aaatCvAUfKttLearuWrP9MDH5MBPbIqV92AaeXatLxBI9gBaebbnrfifHhDYfgasaacH8akY=wiFfYdH8Gipec8Eeeu0xXdbba9frFj0=OqFfea0dXdd9vqai=hGuQ8kuc9pgc9s8qqaq=dirpe0xb9q8qiLsFr0=vr0=vr0dc8meaabaqaciaacaGaaeqabaqabeGadaaakeaacqWGqbaucqGGOaakcqWGfbqrcqGG8baFcqWGRbWAcqGGPaqkcqGH9aqpdaqadaqaaiabigdaXiabgUcaRGGaciab=D7aOnaabmaabaWaaSaaaeaacqWF8oqBaeaacqWF7oaBaaaacaGLOaGaayzkaaWaaWbaaSqabeaacqWGRbWAaaGccqWGLbqzdaahaaWcbeqaaiabgkHiTiabcIcaOiab=X7aTjabgkHiTiab=T7aSjabcMcaPiabdYgaSbaaaOGaayjkaiaawMcaamaaCaaaleqabaGaeyOeI0IaeGymaedaaaaa@4BFD@

How does Equation 8 relate to our retrieval model? Recall from Equation 3 that we need to compute *P*(*c*|*s*_*j*_) and *P *(*d*|*s*_*j*_)–the probability that a user would want to see a particular document given interest in a specific topic. Let us employ *P*(*E*|*k*) for exactly this purpose: we assume that users want to see the elite set of documents for a particular topic, which is computed by observing the frequency of the term that represents the topic. Finally, we approximate *P*(*s*_*i*_) with *idf*, that is, the inverse document frequency of *t*_*i*_. Putting everything together, we derive the following term weighting and document ranking function:

wt=(1+η(μλ)ke−(μ−λ)l)−1idft
 MathType@MTEF@5@5@+=feaafiart1ev1aaatCvAUfKttLearuWrP9MDH5MBPbIqV92AaeXatLxBI9gBaebbnrfifHhDYfgasaacH8akY=wiFfYdH8Gipec8Eeeu0xXdbba9frFj0=OqFfea0dXdd9vqai=hGuQ8kuc9pgc9s8qqaq=dirpe0xb9q8qiLsFr0=vr0=vr0dc8meaabaqaciaacaGaaeqabaqabeGadaaakeaacqWG3bWDdaWgaaWcbaGaemiDaqhabeaakiabg2da9maabmaabaGaeGymaeJaey4kaSccciGae83TdG2aaeWaaeaadaWcaaqaaiab=X7aTbqaaiab=T7aSbaaaiaawIcacaGLPaaadaahaaWcbeqaaiabdUgaRbaakiabdwgaLnaaCaaaleqabaGaeyOeI0IaeiikaGIae8hVd0MaeyOeI0Iae83UdWMaeiykaKIaemiBaWgaaaGccaGLOaGaayzkaaWaaWbaaSqabeaacqGHsislcqaIXaqmaaGcdaGcaaqaaiabdMgaPjabdsgaKjabdAgaMnaaBaaaleaacqWG0baDaeqaaaqabaaaaa@4E06@

Sim(c,d)=∑t=1Nwt,c⋅wt,d
 MathType@MTEF@5@5@+=feaafiart1ev1aaatCvAUfKttLearuWrP9MDH5MBPbIqV92AaeXatLxBI9gBaebbnrfifHhDYfgasaacH8akY=wiFfYdH8Gipec8Eeeu0xXdbba9frFj0=OqFfea0dXdd9vqai=hGuQ8kuc9pgc9s8qqaq=dirpe0xb9q8qiLsFr0=vr0=vr0dc8meaabaqaciaacaGaaeqabaqabeGadaaakeaacqqGtbWucqqGPbqAcqqGTbqBcqGGOaakcqWGJbWycqGGSaalcqWGKbazcqGGPaqkcqGH9aqpdaaeWbqaaiabdEha3naaBaaaleaacqWG0baDcqGGSaalcqWGJbWyaeqaaOGaeyyXICTaem4DaC3aaSbaaSqaaiabdsha0jabcYcaSiabdsgaKbqabaaabaGaemiDaqNaeyypa0JaeGymaedabaGaemOta4eaniabggHiLdaaaa@4A6A@

A term's weight with respect to a particular document (*w*_*t*_) can be computed using Equation 9, derived from the estimation of eliteness in our probabilistic topic similarity model. Similarity between two documents is computed by an inner product of term weights, and documents are sorted by their similarity to the current document *d *in the final output. We note that this derivation shares similarities with existing probabilistic retrieval models, which we discuss in Section 3.

### 1.2 Parameter Estimation

The optimization of parameters is one key to good retrieval performance. In many cases, test collections with relevance judgments are required to tune parameters in terms of metrics such as mean average precision (the standard single-point measure for quantifying system performance in the IR literature). However, test collections are expensive to build and not available for many retrieval applications. To address this issue, we have developed a novel process for estimating *pmra *parameters that does not require relevance judgments.

The *pmra *model has three parameters: *λ*, *μ*, and *η *. The first two define the means of the elite and non-elite Poisson distributions, respectively, and the third is *P*(E¯
 MathType@MTEF@5@5@+=feaafiart1ev1aaatCvAUfKttLearuWrP9MDH5MBPbIqV92AaeXatLxBI9gBaebbnrfifHhDYfgasaacH8akY=wiFfYdH8Gipec8Eeeu0xXdbba9frFj0=OqFfea0dXdd9vqai=hGuQ8kuc9pgc9s8qqaq=dirpe0xb9q8qiLsFr0=vr0=vr0dc8meaabaqaciaacaGaaeqabaqabeGadaaakeaacuWGfbqrgaqeaaaa@2DD7@)/*P*(*E*). To make our model computationally tractable, we make one additional simplifying assumption: that half the term occurrences in the document are elite and the other half are not. This corresponds to assuming a uniform probability distribution in absence of any other information–a similar principle underlies maximum entropy models commonly used in natural language processing [13]. This leads to the following:

η(μλ)=P(E¯)μP(E)λ=1
 MathType@MTEF@5@5@+=feaafiart1ev1aaatCvAUfKttLearuWrP9MDH5MBPbIqV92AaeXatLxBI9gBaebbnrfifHhDYfgasaacH8akY=wiFfYdH8Gipec8Eeeu0xXdbba9frFj0=OqFfea0dXdd9vqai=hGuQ8kuc9pgc9s8qqaq=dirpe0xb9q8qiLsFr0=vr0=vr0dc8meaabaqaciaacaGaaeqabaqabeGadaaakeaaiiGacqWF3oaAdaqadaqaamaalaaabaGae8hVd0gabaGae83UdWgaaaGaayjkaiaawMcaaiabg2da9maalaaabaGaemiuaaLaeiikaGIafmyrauKbaebacqGGPaqkcqWF8oqBaeaacqWGqbaucqGGOaakcqWGfbqrcqGGPaqkcqWF7oaBaaGaeyypa0JaeGymaedaaa@41B8@

Experimental results presented in Sections 2.2 and 2.3 suggest that this assumption works reasonably well. More importantly, it reduces the number of parameters in *pmra *from three to two, and yields a slightly simpler weighting function:

wt=(1+(μλ)k−1e−(μ−λ)l)−1idft
 MathType@MTEF@5@5@+=feaafiart1ev1aaatCvAUfKttLearuWrP9MDH5MBPbIqV92AaeXatLxBI9gBaebbnrfifHhDYfgasaacH8akY=wiFfYdH8Gipec8Eeeu0xXdbba9frFj0=OqFfea0dXdd9vqai=hGuQ8kuc9pgc9s8qqaq=dirpe0xb9q8qiLsFr0=vr0=vr0dc8meaabaqaciaacaGaaeqabaqabeGadaaakeaacqWG3bWDdaWgaaWcbaGaemiDaqhabeaakiabg2da9maabmaabaGaeGymaeJaey4kaSYaaeWaaeaadaWcaaqaaGGaciab=X7aTbqaaiab=T7aSbaaaiaawIcacaGLPaaadaahaaWcbeqaaiabdUgaRjabgkHiTiabigdaXaaakiabdwgaLnaaCaaaleqabaGaeyOeI0IaeiikaGIae8hVd0MaeyOeI0Iae83UdWMaeiykaKIaemiBaWgaaaGccaGLOaGaayzkaaWaaWbaaSqabeaacqGHsislcqaIXaqmaaGcdaGcaaqaaiabdMgaPjabdsgaKjabdAgaMnaaBaaaleaacqWG0baDaeqaaaqabaaaaa@4E3C@

Nevertheless, we must still determine the parameters *λ *and *μ *(Poisson parameters for the elite and non-elite distributions). If a document collection were annotated with actual topics, then these values could be estimated directly. Fortunately, for MEDLINE we have exactly this metadata–in the form of MeSH terms associated with each record. MeSH terms are useful for parameter estimation in our model precisely because they represent topics present in the articles. Thus, we can assume that if *H*_*n *_is assigned to document *d*, the terms in the MeSH descriptor are elite. For example, if the MeSH descriptor "headache" [C10.597.617.470] were assigned to a citation, than the term "headache" must be elite in that abstract. We can record the frequency of the term and estimate *λ *from such observations. Similarly, we can treat as the non-elite case terms in a document that do not appear in any MeSH descriptors, and from this we can derive *μ*. There is, however, one additional consideration: from what set of citations should these parameters be estimated? A few possibilities include: the entire corpus, a random sample, or a biased sample (e.g., results of a search). In this work, we experiment with variants of the third approach.

As a final note, while it is theoretically possible to estimate the parameter *η *based on MeSH descriptors using a similar procedure, this assumes that the coverage of MeSH terms is complete, i.e., that they completely enumerate all topics present in the abstract. Since the assignment of MeSH is performed by humans, we suspect that recall is less than perfect–therefore, we do not explore this idea further.

## 2 Results

### 2.1 Experimental Design

We evaluated our *pmra *retrieval model against *bm25*–a comparison that is appropriate given their shared theoretical ancestry (see Section 3.2). Despite the popularity and performance of language modeling techniques for information retrieval (see [14] for an overview), *bm25 *remains a competitive baseline.

Our experiments were conducted using the test collection from the TREC 2005 genomics track [15], which used a ten-year subset of MEDLINE. The test collection contains fifty information needs and relevance judgments for each, which take the form of lists of PMIDs (unique identifiers for MEDLINE citations) that were previously determined to be relevant by human assessors. See Section 5.1 for more details.

The evaluation was designed to mimic the operational deployment of related article search in PubMed as much as possible. In total, there are 4584 known relevant documents in the test collection from the TREC 2005 genomics track. Each abstract served as a test "query", and we evaluated the top five results under different experimental conditions (the same number that the current PubMed interface shows). Precision, a standard metric for quantifying retrieval performance, is defined as:

Precision=#of relevant documents#of retrieved documents
 MathType@MTEF@5@5@+=feaafiart1ev1aaatCvAUfKttLearuWrP9MDH5MBPbIqV92AaeXatLxBI9gBaebbnrfifHhDYfgasaacH8akY=wiFfYdH8Gipec8Eeeu0xXdbba9frFj0=OqFfea0dXdd9vqai=hGuQ8kuc9pgc9s8qqaq=dirpe0xb9q8qiLsFr0=vr0=vr0dc8meaabaqaciaacaGaaeqabaqabeGadaaakeaacqqGqbaucqqGYbGCcqqGLbqzcqqGJbWycqqGPbqAcqqGZbWCcqqGPbqAcqqGVbWBcqqGUbGBcqGH9aqpdaWcaaqaaiabcocaJiabb+gaVjabbAgaMjabbccaGiabbkhaYjabbwgaLjabbYgaSjabbwgaLjabbAha2jabbggaHjabb6gaUjabbsha0jabbccaGiabbsgaKjabb+gaVjabbogaJjabbwha1jabb2gaTjabbwgaLjabb6gaUjabbsha0jabbohaZbqaaiabcocaJiabb+gaVjabbAgaMjabbccaGiabbkhaYjabbwgaLjabbsha0jabbkhaYjabbMgaPjabbwgaLjabbAha2jabbwgaLjabbsgaKjabbccaGiabbsgaKjabb+gaVjabbogaJjabbwha1jabb2gaTjabbwgaLjabb6gaUjabbsha0jabbohaZbaaaaa@7414@

More specifically, we measured precision at a cutoff of five retrieved documents, commonly written as P5 for short. Since our test collection contains a list of relevant PMIDs for each information need (i.e., the relevance judgments), this computation was straightforward.

We performed two types of experiments:

• a number of runs that exhaustively explored the parameter space to determine optimal values, and

• additional runs of *pmra *using parameters that were estimated in different ways.

The *pmra *experiments used the ranking algorithm described in the previous section. For *bm25*, we used the complete text of the abstract verbatim as the "query" and treated the resulting output as the ranked list of related documents. Finally, as a computational expedient, we ran retrieval experiments as a reranking task using the top 100 documents retrieved by *bm25 *with default parameter settings (*k*_1 _= 1.2, *b *= 0.75), as implemented in the open source Lemur Toolkit for language modeling and information retrieval [16]. Due to the large number of queries involved in our exhaustive exploration of the parameter space and the length of each query (the entire abstract text), this setup made the problem much more tractable given the computational resources we had access to (half a dozen commodity PCs). Since we were only evaluating the top five hits, we believe that this procedure is unlikely to yield different results from a retrieval run against the complete corpus. An experiment to validate this assumption is presented in Section 5.2.

The following procedures were adopted for our exhaustive runs: For *bm25*, we tried all possible parameter combinations, with *k*_1 _ranging from 0.5 to 3.0 in 0.1 increments and *b *from 0.6 to 1.0 in 0.05 increments. This range was selected based on the default settings of *k*_1 _= 1.2, *b *= 0.75 widely reported in the literature. Our exploration of the *pmra *parameter space started with arbitrary values of *λ *and *μ*. Assuming that the performance surface was convex and smooth, we tried different values until its shape became apparent. This was accomplished by first fixing a *λ *value and varying *μ *values in increments of 0.001; this process was repeated for different *λ *values in 0.001 increments.

In the second set of experiments, *λ *and *μ *for *pmra *were estimated using the procedure described in Section 1.2, on different sets of citations. We also performed cross-validation as necessary to further verify our experimental results.

### 2.2 Optimal Parameters

**Figure 2:**
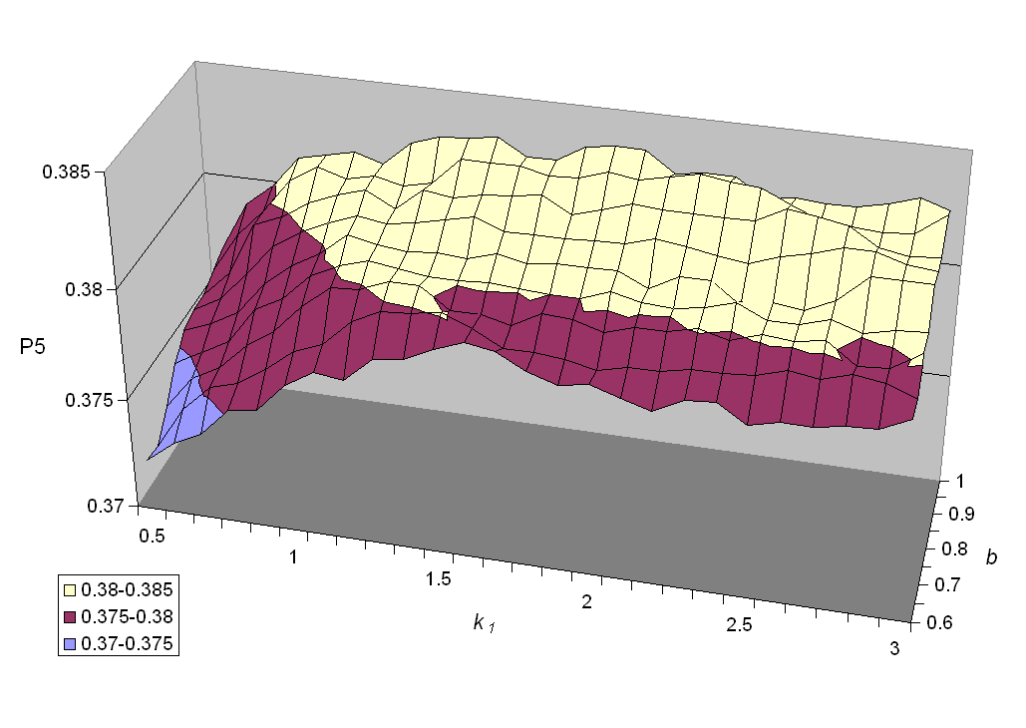
P5 for the *bm25 *model given different settings of the parameters *k*_1 _and *b*. This plot was generated by exhaustively trying all *k*_1 _values 0.5 to 3.0 (in 0.1 increments) and *b *values 0.6 to 1.0 (in 0.05 increments). Notice that except for low values of *k*_1 _and *b*, P5 performance is relatively insensitive to parameter settings.

**Figure 3:**
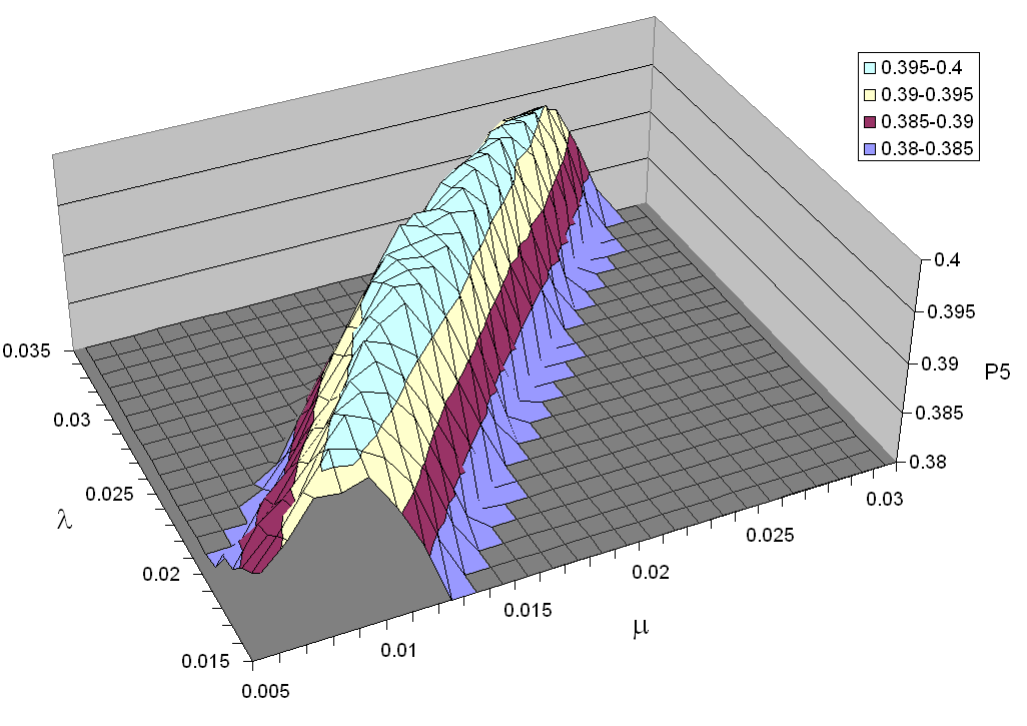
P5 for the *pmra *model given different settings of the parameters *λ *(Poisson parameter for the elite distribution) and *μ *(Poisson parameter for the non-elite distribution). Notice that the parameter settings resulting in high P5 values lie along a "ridge" in the parameter space.

The results of our exhaustive parameter tuning experiments for *bm25 *are shown in Figure 2, which plots precision at five across a wide range of parameter values. We note that except for low values of *k*_1 _and *b*, P5 performance is relatively insensitive to parameter settings (more on this below). Results for the *pmra *parameter tuning experiments are shown in Figure 3–regions in the parameter space that yield high precision lie along a prominent "ridge" that cuts diagonally from smaller to larger values of *λ *and *μ *(more on this in Section 3.3).

The highest P5 performance for *bm25 *is achieved with *k*_1 _= 1.9 and *b *= 1.0; by the same metric, the optimal setting for *pmra *is *λ *= 0.022 and *μ *= 0.013. Table 1 shows precision at five values numerically for optimal *bm25 *and optimal *pmra*, which we refer to as bm25* and pmra* for convenience. For comparison, the performance of *bm25 *with default parameter values *k*_1 _= 1.2, *b *= 0.75 (denoted as *bm25*^b^) is also shown. We applied the Wilcoxon signed-rank test to determine if the differences in the evaluation metrics are statistically significant. Throughout this paper, significance at the 1% level is indicated by **; significance at the 5% level is indicated by *. Differences that are not statistically significant are marked with the symbol °. Results show a small, but statistically significant improvement of *pmra *over *bm25 *(both default and optimized), but no significant difference between optimized and default *bm25*. Due to the large number of test abstracts, we are able to discriminate small differences in performance between the models (recall that each of the 4584 relevant documents from the test collection was used as a test abstract).

**Table 1:** Overall comparison between the *bm25 *and *pmra *models.

**Run**	**Model**	**Description**	**P5**	*vs. bm25*^b^	bm25*
*bm25*^b^	*bm25 *(*k*_1 _= 1.2, *b *= 0.75)	*bm25*, default parameters	0.381		-0.5%°
bm25*	*bm25 *(*k*_1 _= 1.9, *b *= 1.00)	*bm25*, optimal parameters	0.383	+0.5%°	
pmra*	*pmra *(*λ *= 0.022, *μ *= 0.013)	*pmra*, optimal parameters	0.399	+4.7% **	+4.2% **

Table shows model parameters, P5 values over the entire test collection, and relative differences.

Information needs from the TREC 2005 genomics track were grouped into five templates, each with ten different instantiations; see Section 5.1 for more details. Precision at five values broken down by template are shown in Table 2. Relative differences are shown in Table 3, along with the results of Wilcoxon signed-rank tests. We find that in general, differences between default and optimized *bm25 *are not statistically significant, except for template #3. Optimized *pmra *outperforms optimized *bm25 *on four out of five templates, three of which are statistically significant.

**Table 2:** Comparison between the *bm25 *and *pmra *models, broken down by template.

**Template**	*bm25*^b^	bm25*	pmra*
#1: methods or protocols	0.211	0.210	0.253
#2: role of gene in disease	0.484	0.487	0.499
#3: role of gene in biological process	0.351	0.365	0.349
#4: gene interactions in organ/disease	0.297	0.281	0.303
#5: mutation of gene and its impact	0.440	0.438	0.462

Table shows P5 values for *bm25*^b ^(default parameters), bm25* (optimized parameters), and pmra* (optimized parameters).

**Table 3:** Relative differences between the *bm25 *and *pmra *models.

**Template**	bm25* *vs. bm25*^b^	pmra* *vs. bm25*^b^	pmra* vs. bm25*
#1: methods or protocols	-0.5%°	+20.0% **	+20.5% **
#2: role of gene in disease	+0.6%°	+3.1% *	+2.5% *
#3: role of gene in biological process	+4.0% **	-0.6%°	-4.4% **
#4: gene interactions in organ/disease	-5.4%°	+2.0%°	+7.8%°
#5: mutation of gene and its impact	-0.5%°	+5.0% *	+5.5% **

Three conditions are compared: *bm25*^b ^(default parameters), bm25* (optimized parameters), and pmra* (optimized parameters). Each column represents a *x vs. y *comparison, where the figures indicate the relative improvements of *x *over *y*. In general, we see that the differences between optimized and default *bm25 *are not statistically significant, whereas the differences between *pmra *and *bm25 *are.

### 2.3 Estimated Parameters

We also attempted to automatically estimate parameters for the *pmra *model using the method described in Section 1.2. However, that method is underspecified with respect to the set of MEDLINE citations over which it is applied. We experimented with the following possibilities:

• The complete set of documents examined by human assessors in the TREC 2005 genomics track (see [3] for a description of how these documents were gathered).

• The top 100 hits for each of the 4584 PMIDs that comprise our test abstracts, using *bm25 *with default parameters.

• The top 100 hits for each of the 50 template queries that comprise the TREC 2005 genomics track, retrieved using Indri's default ranking algorithm based on language models. Indri is a component in the open source Lemur Toolkit.

• Same as previous, except with top 1000 hits.

The estimated parameters given each citation set is shown in Table 4, along with the size of each set and the precision achieved. In the first condition, the estimated parameters differ from the optimal ones, but the resulting P5 figure is statistically indistinguishable. For the three other citation sets, the estimated parameters were very close to the optimal parameters. Once again, the differences are not statistically significant. These results suggest that our parameter estimation method is robust and effective. Furthermore, it also appears to be insensitive with respect to the size and composition of the citation set.

**Table 4:** Values of *pmra *parameters (*λ*, *μ*) estimated using different sets of MEDLINE citations.

**Set Used**	**Size**	*λ*	*μ*	**P5**
All assessed documents from TREC 2005 genomics track	39874	0.032	0.022	0.397°
Top 100 hits for every relevant citation, *bm25*	453402	0.023	0.013	0.398°
Top 100 hits for every template query, Indri	4991	0.022	0.012	0.397°
Top 1000 hits for every template query, Indri	49907	0.024	0.013	0.397°
Optimal parameters		0.022	0.013	0.399

We see that estimated values are close to optimal parameter values in many cases, and that differences in P5 performance are not statistically significant.

Finally, to further verify these results and to ensure that we were not estimating parameters from the same set used to measure precision, cross-validation experiments were performed on the second condition. The 4584 test abstracts were divided into five folds, stratified across the templates so that each template was represented in each fold. We conducted five separate experiments, using four of the folds for parameter estimation and the final fold for evaluation. The results were exactly the same–P5 figures were statistically indistinguishable from the optimal values.

In summary, we have empirically demonstrated the effectiveness of our *pmra *retrieval model and shown a small but statistically significant improvement in precision at five documents over the *bm25 *baseline. Furthermore, our novel parameter estimation method was found to be effective when applied to a wide range of citation sets varying in both composition and size. Notably, the tuning of parameters did not require relevance judgments, the component in a test collection that is the most expensive and time-consuming to gather.

## 3 Discussion

### 3.1 Significance of Results

Although we measured statistically significant differences in P5 between *pmra *and *bm25*, are the improvements meaningful in a real sense? The difference between baseline *bm25 *and optimal *pmra *(achievable by our parameter estimation process) is 4.7%. In terms of the PubMed interface, for each abstract, one would expect 2.0 *vs. *1.9 interesting articles in the related links display. We argue that although small, this is nevertheless a meaningful improvement.

PubMed is one of the Internet's most-visited gateways to MEDLINE–small differences, multiplied by thousands of users and many more interactions add up to substantial quantities. In addition, our metrics are measuring performance differences *per interaction*, since a list of related articles is retrieved for every citation that the user examines. In the course of a search session, a user may examine many citations, especially when conducting in-depth research on a particular subject. Thus, the effects of small performance improvements accumulate.

One might also argue that this accumulation of benefits is not linear. Consider the case of repeatedly browsing related articles–the user views a citation, examines related articles, selects an interesting one, and repeats (cf. the simulation studies in [17,18]). In that case, the expected number of interesting links per interaction can be viewed as a branching factor if one wanted to quantify the total number of interesting articles that are accessible in this manner. In about 13 interactions, an improvement of 0.1 (i.e., 1.9^13 ^vs. 2.0^13^) would result in potential access to twice as many interesting articles.

### 3.2 Comparison to Other Work

A suitable point of comparison for this work is the Binary Independent Retrieval (BIR) model for probabilistic IR [5,6], which underlies *bm25*. Indeed, *bm25 *was chosen as a baseline not only for its performance, but also because it shares certain theoretical similarities with our model. Along with related work dating back several decades [8,9], these two models share in their attempts to capture term frequencies with Poisson distributions. However, there are important differences that set our work apart.

The *pmra *model was designed for a fundamentally different task–related document search, not *ad hoc *retrieval. In the latter, the system's task is to return a ranked list of documents that is relevant to a user's query (what most people think of as "search"). One substantial difference is query length–in *ad hoc *retrieval, user queries are typically very short (a few words at the most). As a result, query-length normalization is not a critical problem, and hence has not received much attention. In contrast, since the "query" in related document search is a complete document, more care is required to account for document length differences.

Another important difference between *pmra *and *bm25 *is that there is no notion of relevance in the *pmra *model, only that of relatedness, mediated via topic similarity. Note, however, that the concept of relevance is still *implicitly *present in the task definition–in that the examination of documents may take place in the context of broader information-seeking behaviors. In contrast, the starting point of BIR is a log-odds, i.e., *P*(*R*|*D*)/*P*(R¯
 MathType@MTEF@5@5@+=feaafiart1ev1aaatCvAUfKttLearuWrP9MDH5MBPbIqV92AaeXatLxBI9gBaebbnrfifHhDYfgasaacH8akY=wiFfYdH8Gipec8Eeeu0xXdbba9frFj0=OqFfea0dXdd9vqai=hGuQ8kuc9pgc9s8qqaq=dirpe0xb9q8qiLsFr0=vr0=vr0dc8meaabaqaciaacaGaaeqabaqabeGadaaakeaacuWGsbGugaqeaaaa@2DF1@|*D*), which explicitly attempts to estimate the relevance (*R*) and non-relevance (R¯
 MathType@MTEF@5@5@+=feaafiart1ev1aaatCvAUfKttLearuWrP9MDH5MBPbIqV92AaeXatLxBI9gBaebbnrfifHhDYfgasaacH8akY=wiFfYdH8Gipec8Eeeu0xXdbba9frFj0=OqFfea0dXdd9vqai=hGuQ8kuc9pgc9s8qqaq=dirpe0xb9q8qiLsFr0=vr0=vr0dc8meaabaqaciaacaGaaeqabaqabeGadaaakeaacuWGsbGugaqeaaaa@2DF1@) of a document (*D*). Relevance is then modeled in terms of eliteness (see below). The starting point of our task definition leads to a different derivation.

Although both *bm25 *and *pmra *attempt to capture term dependencies in terms of Poisson distributions, they do so in different ways. BIR employs a more complex representation, where term frequencies are modeled as mixtures of two different Poisson distributions (elite and non-elite). In total, the complete model has four parameters–the two Poisson parameters, *P*(*E*|*R*), and *P*(*E*|R¯
 MathType@MTEF@5@5@+=feaafiart1ev1aaatCvAUfKttLearuWrP9MDH5MBPbIqV92AaeXatLxBI9gBaebbnrfifHhDYfgasaacH8akY=wiFfYdH8Gipec8Eeeu0xXdbba9frFj0=OqFfea0dXdd9vqai=hGuQ8kuc9pgc9s8qqaq=dirpe0xb9q8qiLsFr0=vr0=vr0dc8meaabaqaciaacaGaaeqabaqabeGadaaakeaacuWGsbGugaqeaaaa@2DF1@). Since eliteness is a hidden variable, there is no way to estimate the parameters directly. Instead, Robertson and Walker devised simple approximations that work well empirically [19]. One side effect of this 2-Poisson approximation is that *bm25 *parameters are not physically meaningful, unlike *λ *and *μ *in *pmra*, which correspond to comprehensible quantities. Unlike BIR, our model makes the simplifying assumption that terms are exclusively drawn from either the elite or non-elite distribution. That is, if the document is about a particular topic, then the corresponding term frequency is dictated solely by the elite Poisson distribution; similarly, the non-elite distribution for the non-elite case.

Finally, the derivation of our model, coupled with the availability of MeSH headings in the biomedical domain, allow us to directly estimate parameters for our system. Most notably, the process does not require a test collection with relevance judgments, making the parameter optimization process far less onerous.

### 3.3 Parameter Estimation

The estimation of parameters in the *pmra *model depends on the existence of MeSH terms, which is indeed a fortuitous happenstance in the case of MEDLINE. Does this limit the applicability of our model to other domains in which topic indexing and controlled vocabularies are not available? We note that effective access to biomedical text is suffciently important an application that even a narrowly-tailored solution represents a contribution. Nevertheless, we present evidence to suggest that the *pmra *model provides a general solution to related document search.

**Figure 4:**
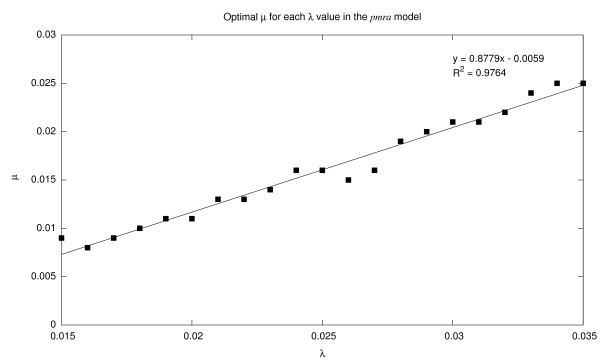
Optimal *μ *(Poisson parameter for the non-elite distribution) for each *λ *value (Poisson parameter for the elite distribution) in the *pmra *model. Regression line shows a linear relationship between these two parameters, corresponding to the "ridge" in Figure 3.

We see from Figure 3 that our model performs well with settings that lie along a ridge in the parameter space. This observation is confirmed in Figure 4–for each value of *λ *(from 0.015 to 0.035 in increments of 0.001), we plot the optimal value of *μ*. Superimposed on this graph is a linear regression line, which achieves an *R*^2^ value of 0.976, a very good fit. This finding suggests that the relationship between *λ *and *μ *is perhaps even more important than their absolute values, since good performance is attainable with a wide range of parameter settings (as long as the relationship between *λ *and *μ *is maintained).

**Figure 5:**
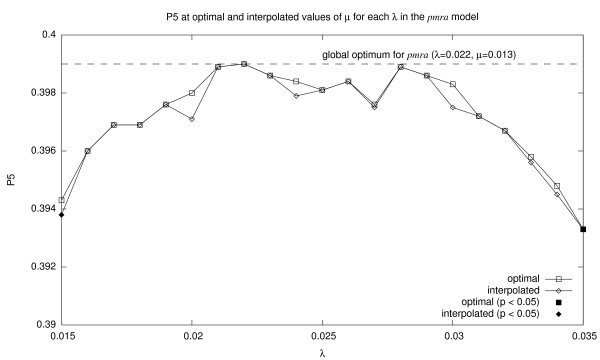
P5 at optimal and interpolated values of *μ *for each *λ *in the *pmra *model. Squares represent optimal *μ *at each *λ*, corresponding to the squares in Figure 4. Diamonds represent interpolated *μ *at each *λ*, corresponding to the regression line in Figure 4. P5 of the globally optimal parameter setting is shown as the dotted line. The filled square and diamond represent points at which P5 is significantly lower than the globally optimal setting.

How good is related document search performance along this ridge? The answer is found in Figure 5. On the *x*-axis we plot values of *λ *; the *y*-axis shows P5 values for two conditions–optimal *μ *(for that *λ*), shown as squares, and interpolated *μ *based on the regression line in Figure 4, shown as diamonds. The performance of the globally-optimal setting (*λ *= 0.022, *μ *= 0.013, which yields P5 = 0.399) is shown as the dotted line. We see that across a wide range of parameter settings, P5 performance remains close to the global optimum. The Wilcoxon signed-rank test was applied to compare the performance at each setting with the globally-optimal setting: differences that are statistically significant (*p *< 0.05) are shown as solid diamonds and squares. Only at both ends of the wide *λ *range do differences become significant.

This finding suggests that the *pmra *model is relatively insensitive to parameter settings, so long as a particular relationship is maintained between *λ *and *μ*. Thus, it would be reasonable to apply our model to texts for which controlled-vocabulary resources do not exist.

## 4 Conclusion

In most search applications, system input is comprised of a short query, which is a textual representation of the user's information need. In contrast, this work focuses on related document search, where given a document, the goal is to find other documents that may be of interest to the user–in our case, the specific task is to retrieve related MEDLINE abstracts. We present a novel probabilistic topic-based content similarity algorithm for accomplishing this, deployed in the PubMed search engine. Experiments on the TREC 2005 genomics track test collection show a small but statistically significant improvement over *bm25*, a competitive probabilistic retrieval model. Evidence suggests that the *pmra *model is able to effectively retrieve related articles, and that its integration into PubMed enriches the user experience.

## 5 Methods

### 5.1 Test Collection

The test collection used in our experiments was developed from the TREC 2005 genomics track [15]. The Text Retrieval Conferences (TRECs) are annual evaluations of information retrieval systems that draw dozens of participants from all over the world each year [20]. Numerous "tracks" at TREC focus on different aspects of information retrieval, ranging from spam detection to question answering. The genomics track in 2005 focused on retrieval of MEDLINE abstracts in response to typical information needs of biologists and other biomedical researchers.

The live MEDLINE database as deployed in PubMed is constantly evolving as new articles are added, making it unsuitable for controlled, reproducible experiments. Therefore, the TREC 2005 genomics track evaluation employed a ten-year subset of MEDLINE (1994–2003), which totals 4.6 million citations (approximately a third of the size of the entire database at the time it was collected in 2004). Each record is identified by a unique PMID and includes bibliographic information and abstract text (if available).

One salient feature of the evaluation is its use of generic topic templates (GTTs) to capture users' information needs, instead of the typical free-text title, description, and narrative combinations used in other *ad hoc *retrieval tasks, e.g., [21]. The GTTs consist of semantic types, such as genes and diseases, that are embedded in common genomics-related information needs, as determined from interviews with biologists. In total, five templates were developed, with ten fully-instantiated information needs for each; examples are shown in Table 5. The templates impose a level of organization on the information needs, but do not have a substantial impact on system performance since participants for the most part did not exploit the template structure, but instead treated the topics no differently than free-text queries.

**Table 5:** Templates and sample instantiations used in the TREC 2005 genomics track evaluation.

#1 **Information describing standard **[**methods or protocols**] **for doing some sort of experiment or procedure.**
*methods or protocols: *how to "open up" a cell through a process called "electroporation"
#2 **Information describing the role(s) of a **[**gene**] **involved in a **[**disease**].
*gene: *interferon-beta
*disease: *multiple sclerosis
#3 **Information describing the role of a **[**gene**] **in a specific **[**biological process**].
*gene: *nucleoside diphosphate kinase (NM23)
*biological process: *tumor progression
#4 **Information describing interactions between two or more **[**genes**] **in the **[**function of an organ**] **or in a **[**disease**].
*genes: *CFTR and Sec61
*function of an organ: *degradation of CFTR
*disease: *cystic fibrosis
#5 **Information describing one or more **[**mutations**] **of a given **[**gene**] **and its **[**biological impact or role**].
*gene with mutation: *BRCA1 185delAG mutation
*biological impact: *role in ovarian cancer

In total, 32 groups submitted 59 runs to the TREC 2005 genomics track, consisting of both automatic runs and those with human intervention. Relevance judgments were provided by an undergraduate student and a Ph.D. researcher in biology. We adapted the judgments for our task by treating each relevant document as a test abstract–citations relevant to the same information need were said to be related to each other. In other words, we assume that if a user were examining a MEDLINE citation to address a particular information need, other relevant citations would also be of interest.

### 5.2 Reranking Experiments

Recall from Section 2.1 that for computational expediency, our experiments were performed as reranking runs over results retrieved by *bm25 *with default paramters. We describe an experiment that examined the potential impact of this setup.

In theory, both *bm25 *and *pmra *establish an ordering over *all *documents in a corpus with respect to a query. Reranking in the limit yields exactly the same results; thus, the substantive question is whether reranking the top hundred hits would yield the same results as searching over the entire corpus. We can examine this issue by tallying the original rank positions of the top five results after reranking–that is, if reranking promotes hits that are highly ranked in the original list to begin with, then we can conclude that hits in the lower ranked positions of the original list matter little. On the other hand, if the reranking brings up hits that are very far down in the original ranked list, it might cause us to wonder what other documents from lower-ranked positions are missed.

**Figure 6:**
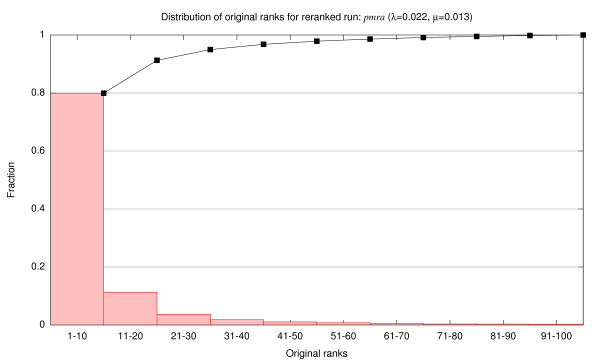
Distribution of original ranks for reranked run: *pmra *(*λ *= 0.022, *μ *= 0.013). The bar graph divides the original rank positions into ten bins and tallies the fraction of hits that were brought into the top five by *pmra*; for example, approximately 80% of the top five *pmra *results came from the top ten results in the original ranked list. The line graph shows the cumulative distribution.

We performed exactly this experiment with the optimal *pmra *run (*λ *= 0.022, *μ *= 0.013). For each test abstract, we tallied the original ranks of the top five results, e.g., hit 1 of *pmra *was promoted from hit 9 of the original ranked list, etc. We divided the original rank positions into ten bins of equal size and plotted a histogram of the bin frequencies. The results are shown by the bar graph in Figure 6; the line graph shows the corresponding cumulative distribution. We see, for example, that approximately 80% of the top five *pmra *results came from the top ten results in the original ranked list. That is, 80% of the time the *pmra *algorithm was merely reshuffing the top ten *bm25 *results–this is not unexpected, since *bm25 *already performs well and there's not much to be done in terms of improving the results in many cases. The cumulative distribution tops 95% at rank 31 and 99% at rank 67–which means that *pmra *is promoting hits below these ranks to the top five positions only five and one percent of the time, respectively. Thus, it is unlikely that our reranking setup resulted in different conclusions than if the retrieval had been performed on the entire corpus. This experiment supports the validity of our experimental design.

## Authors' contributions

WJW developed the original *pmra *model. JL worked on subsequent refinements, including the parameter estimation method. JL ran the experiments. Both authors read and approved the final manuscript.
